# Factors associated with retakes in health Professions Courses: A case study of Five selected Universities in Sub-Saharan Africa

**DOI:** 10.21203/rs.3.rs-5368416/v1

**Published:** 2024-11-19

**Authors:** RONALD KIBUUKA, Ferastas Mpasa, Irene Atuhairwe, Brian Agaba, Prossy Nakattudde, Samuel Owusu-Sekyere, Abigail Amponsah, Ndikom Chizoma, Ogah Oluwakemi, Kiyimba Kennedy, Obakiro Samuel Baker, Atipasta Kaminga, Joshua Epuitai, Etta Chimbe, Masumbuko Baluwa, Getrude Munthali, Katuramu Richard

**Affiliations:** Busitema University faculty of Health Sciences, Department of Pharmacology and Therapeutics; Mzuzu University; Seed Global Health; Seed Global Health; Seed Global Health; African Forum for Research and Education in Health; Kwame Nkrumah University of Science and Technology; University of Ibadan; University of Zambia School of Medicine; Busitema University faculty of Health Sciences, Department of Pharmacology and Therapeutics; Busitema University faculty of Health Sciences, Department of Pharmacology and Therapeutics; Mzuzu University; Busitema University Faculty of Health Sciences, Department of Nursing; Mzuzu University; Mzuzu University; Mzuzu University; Busitema University faculty of Health Sciences, Department Internal Medicine

**Keywords:** retakes, health professions education, Sub-Saharan Africa, OSCE

## Abstract

**Background:**

Academic examination retakes are significant challenges in health professions education. With rigorous clinical assessments, limited training resources and high-stakes examinations, students struggle to meet academic requirements which cab result in retakes. This study aimed to assess the proportion of medical and nursing students with retakes across five Sub-Saharan African universities and to explore factors contributing to the retakes.

**Methods:**

This was a cross-sectional quantitative study. The study involved 764 medical and nursing clinical students from five universities across Sub Saharan Africa: Busitema University (Uganda), Mzuzu University (Malawi), University of Ibadan (Nigeria), Kwame Nkrumah University of Science and Technology (Ghana), and the University of Zambia. Data was analyzed using descriptive statistics and logistic regression to identify factors associated with retakes.

**Results:**

Overall, the proportion of students who had ever had a retake in clinical assessments was 12.6%. Factors associated with retakes are age with students aged 25 and above showed a higher likelihood of failing compared to their younger counterparts (OR: 2.45, 95% CI: 1.06–5.64, *P* = 0.036) and gender with Male students more likely to get retakes compared to their female counterparts (OR: 1.91, 95% CI: 1.21–3.00, *P* = 0.005) at univariate, although this association was not statistically significant in multivariate analysis. OSCE was the most frequent examination format associated with retakes, accounting for 47.7% (*P* < 0.001).

**Conclusions:**

The present study brings to light the proportion of students who experienced retakes and the factors associated with retakes among health professions students in Sub-Saharan Africa. This highlights the need for institutional interventions targeting at-risk populations: older and male students, and those with assessment problems related to the different modes of assessing clinical skills. Amelioration of these factors through appropriate support systems could minimize the proportion of students having to retake modules and create a more supportive academic environment. Further research might focus on exploring the proportion of students experiencing retakes in each country and thus determine factors that could contribute to high stakes towards retakes for each country so as to develop country specific solution and improved clinical skills assessment.

## Introduction

The journey through medical and nursing education is indeed faced with challenges, particularly during the clinical years, where students face high-stakes examinations and the transition from preclinical to clinical training (Mennin, 2021). The clinical years of health professions education are often associated with a notable incidence of retakes. From the Busitema University academic handbook 2020/21–2023/24 a retake is defined as the act of a student repeating a course or courses when next offered to achieve the pass mark (50%) if they failed during the first assessment or to improve a low pass grade, with the original grade being retained on the transcript if no improvement is made (Busitema University, 2021). However, in this study a retake only refers to a student failing to achieve the pass mark (50%). Studies have shown that the average proportions of students getting retakes in undergraduate health professions education is around 10% (Rashid et al., 2022; Ratnapalan & Jarvis, 2020).

Literature reveals that the retakes in medical education can be attributed to various factors, including inadequate preparation, personal challenges, and mental health issues. A study at a UK university suggests that approximately 10–15% of medical students may fail to progress satisfactorily, which can have long-term implications for their professional practice (Patel et al., 2015). A retake can significantly impact students’ academic paths and mental well-being. Moreover, the traditional remediation strategies often focus on merely passing assessments rather than addressing the underlying issues that contribute to retakes. This approach has been criticized for potentially perpetuating the cycle of retakes rather than fostering genuine understanding and improvement (Ellaway et al., 2018a; Patel et al., 2015).

Despite these challenges, limited research exists on the factors contributing to retakes in health professions education in sub-Saharan Africa. Understanding these factors is essential for designing targeted remediation strategies that could reduce retake occurrences, particularly in resource-limited settings such as Sub-Saharan Africa. Therefore, this study seeks to determine the proportion of students who have ever experienced retake and investigate the factors associated with retakes in health professions programs across five selected universities in sub-Saharan Africa. This knowledge will provide a foundation for interventions that enhance academic support and promote long-term professional success for health professions students in the region with improved clinical skills assessment.

## Methodology

### Study Design

We used a cross-sectional quantitative research design.

### Study Setting and Participants

The study was conducted across five health training Universities in sub–Saharan Africa: Busitema University (Uganda), Mzuzu University (Malawi), University of Ibadan (Nigeria), Kwame Nkrumah University of Science and Technology (Ghana), and the University of Zambia (Zambia).

### Mzuzu University, commonly known as MZUNI, Malawi:

Chartered in 1997 under Chap. 30:09 of the Laws of Malawi, Mzuzu University is a public institution of higher learning committed to fostering knowledge and offering quality education, research, and training. The university aims to meet the educational expectations for Malawi, Africa, and the global community. Courses offered include, from Certificate to Doctorate, and are divided into six faculties that encompass Health Sciences, Education, and Environmental Sciences. It is a dual-mode institution offering face-to-face and Open Distance e-Learning. In addition, over 50 research projects have so far been successfully executed within the university with major funding from international organizations such as the World Bank, ACIAR, and USAID. These projects include, among others, ICT, climate change, health, and renewable energy studies.

### Busitema University, Uganda

Busitema University is a multi-campus public university in Eastern Uganda. It was established in 2007 by Statutory Instrument No. 22 under the Universities and Other Tertiary Institutions Act. Its mission is to provide high-standard training and engage in research that drives socio-economic transformation and sustainable development. Busitema University holds a commitment to academic excellence as it contributes towards the greater development activities of Uganda through research and community outreach.

**The University of Ibadan, Nigeria** was established in 1948 initially as a college of the University of London, then becoming a full-fledged independent university in 1962. Largely recognized as Nigeria’s premier university and its first, the mission of the university is “to expand the frontiers of knowledge through academic excellence geared towards meeting the needs of society.”. UI’s College of Medicine is the first in Nigeria and was established in 1948, followed by the pioneering Nursing program in 1965.

### Kwame Nkrumah University of Science and Technology, Ghana

KNUST was founded in 1951 as the Kumasi College of Technology and became a full-fledged university in 1952; it was later renamed Kwame Nkrumah University of Science and Technology. The vision for this university is that it should be among the leading science and technology institutions in Africa, driving the creation and utilization of knowledge through research, entailing quality teaching and learning, and community involvement through outreach. KNUST commits itself to the promotion of innovation, entrepreneurship, and technological leadership in all its academic and research programs.

### University of Zambia, Zambia

The University of Zambia was established in 1965 and is the largest and oldest learning institution in Zambia. The University officially opened its doors in 1966. UNZA provides education through the medium of English and lies at the heart of Zambia’s education and research. It is one of the most important universities, particularly in training in the areas of health and medical sciences, in furthering academic development both nationally and regionally.

The study sites were selected purposively because they were part of the joint-funded project by AFREhealth small grants Cohort 2 (2023) and also ensuring African regional representation. They have different faculties which include Faculty of engineering, faculty of biological sciences, faculty of education, faculty of agriculture and faculty of health sciences. We then chose students who were in the faculty of health sciences offering Bachelor of medicine,Bachelor of Surgery and Bachelor of Nursing

### Study population

The study population consisted of final year clinical healthcare professional students. Students offering a bachelor of medicine and surgery and bachelor of science in nursing were included in the study. The included students had been exposed to several clinical assessment formats multiple times, Final year students were selected because they had sufficient experience with clinical assessments thus could provide meaningful insights.

### Sampling method

#### Estimation of Sample Size

Since the target population is finite, the following formula) (Krejcie & Morgan, 1970) was used to determine the sample size (Abdul et al., 2021). This formula provides a systematic way of determining the appropriate sample size in relation to the total population to ensure an adequate level of precision in statistical analysis. The formula takes into consideration population size, desired confidence level, and margin of error.

Finite population: $$CI^{\prime }=\hat{p}\pm \text{z}\times \sqrt{\frac{\hat{p}(1-\hat{p})}{n^{\prime }}\times \frac{N-n^{\prime }}{N-1}}$$

Where:

z is z score

$\hat{p}$ is the population proportion

n and n’ are sample size

N is the population size

The final sample size for this study was 571 students, as shown in the Table 1 below:

### Study variables

This study examined the following key variables in the analyses of factors associated with academic retakes among final-year health professions students: The dependent variable was the occurrence of a retake, a variable that captured whether students had experienced a retake during their clinical assessments. Independent variables included demographic and academic factors. These ranged from institution of enrollment, such as Mzuzu University, Busitema University, University of Zambia, Kwame Nkrumah University of science and technology or the University of Ibadan; program of study-in nursing, or medicine and surgery and gender. Other variables were the age of the students, which was grouped into categories like 18–24, 25–34, and 35 years and above. Another variable assessed was the mode of assessment, in which the formats of clinical assessment the students had gone through were typed as OSCE, long case, or short case. Lastly, entry method into their programs, whether through direct entry, upgrading, or alternative pathways, was tested for its effects on occurrences of retakes. These variables outlined patterns and trends that contributed to academic retakes across diverse students.

### Study procedure

A standardized questionnaire was self-administered to all 764 participants in order to collect data on demographic characteristics, academic background, and any history of retakes during clinical years. Other questions included the mode of examination: OSCE, long case, short case, among others, and the type of program the students enrolled in, whether medicine or nursing. The instrument was shared online via email and institutional platforms, and responses were collected over four weeks in March 2024.

### Statistical analysis

Data was analyzed using descriptive statistics to summarize the demographic characteristics of the participants. Retake were calculated as percentages, and these proportions were compared across universities. Further, univariate and multivariate logistic regression analyses were conducted to identify factors associated with retakes, including gender, age, university, and program type. A p-value of < 0.05 was considered statistically significant. All analyses were performed using SPSS version 25.

### Ethical Considerations

The study received ethical approval from Busitema University Research ethics committee BUFHS-2023–132 and administrative clearance from the institutional review boards of the participating universities. Prior to commencement of the study, a memorandum of understanding was signed by the participating institutions. Informed consent was obtained from all participants by having respondents respond to a question on whether they are willing to participate in hthe study selection of **NO** was set to automatically end the questionnaire, ensuring confidentiality and the right to withdraw from the study at any time. All survey responses and interview transcripts were anonymized to protect participants’ identities.

## RESULTS

We approached 851 students and recruited 764 students. As shown in table 2 below. A total of 764 participants were included in the study. More than half of the participants were female (61.9%). Majority of the participants were Christians (90.4%), followed by Muslims (5.8%). Participants’’ ages ranged from 18 to 48 years, with a mean age of 25.38 ± 5.00 years. over a half of the participants (54.5%) were aged 18–24 years. The largest proportions of participants were enrolled in nursing programs (64.5%), and a significant majority (65.4%) entered their programs through direct entry. All participants were pursuing bachelor’s degrees.

### Have you ever failed a paper in your clinical years?

Table 3 summarize the proportion of students who reported ever having a retake during clinical evaluation. The data shows significant differences across the institutions, with Mzuzu University and the University of Zambia having higher rates of students who had retakes compared to other universities. The overall failure rate was 12.6%, but the rates varied significantly by institution (p < 0.001). Kwame Nkrumah University had the lowest failure rate (2.5%), while Mzuzu University reported the highest failure rate (22.1%). Students at Busitema University and the University of Zambia also reported relatively high failure rates at 12% and 14.7%, respectively.

Table 4 illustrates the mode of examination associated with student retakes, showing significant differences by institution (p < 0.001 for most universities). The OSCE (Objective Structured Clinical Examination) was the most common examination format linked to retakes, contributing to 47.7% of failures overall. In Mzuzu University, 81.4% of students who had retakes reported OSCE as the examination mode responsible for their failure. In contrast, at University of Zambia from Zambia, a combination of different assessment methods was more commonly associated with retakes.

From Table 5 the univariate logistic regression analysis shows that Male students had statistically significantly higher odds of experiencing retakes compared to females (COR: 1.91, 95% CI: 1.21–3.0, *P* = 0.005). Students aged 18–24 were less likely to have retakes than older students (COR 0.2 (95% CI: 0.1–0.43, *P* < 0.001). Across universities, students from the University of Ibadan (OR: 0.37, 95% CI: 0.16–0.86, *P* = 0.021) and Kwame Nkrumah University (COR: 0.09, 95% CI: 0.03–0.31, *P* < 0.001) had significantly lower odds of retakes.

In Table 6 the multivariate logistic regression analysis shows that, students aged 35 and above had higher odds of experiencing retakes than the younger counterparts (AOR: 2.45, 95% CI: 1.06–5.64, *P* = 0.036). At Mzuzu University, students aged 18–24 had significantly lower odds of retakes (AOR: 0.2, 95% CI: 0.07–0.61, *P* = 0.005). Regarding institutions, students at Kwame Nkrumah University had significantly lower odds of retakes

## Discussion

The study aimed to assess the proportion of final year nursing and medical students with retakes across five universities in Sub-Saharan Africa. The findings revealed significant institutional variations, with certain student demographics and assessment modes influencing the likelihood of academic retakes. This discussion critically interprets these findings and contextualizes them within the broader literature on medical and health professions education.

### Failure and Retake Rates

The overall proportion of medical and nursing students who had ever had a retake in clinical assessments was found to be 12.6%, with significant differences across institutions. Notably, Kwame Nkrumah University reported the lowest proportion (2.5%), while Mzuzu University recorded the highest (22.1%). Institutional variations can thus be explained by differences in educational systems, teaching quality, or assessment practices. For example, some universities may give more academic support, or their grading system may be less strict, which can account for the low retake occurrences. On the contrary, some universities adopt a multi-layered assessment with less effective support system, hence more students fail. For example, Mzuzu University does an extra licensing exam, which could be the reason behind the high rate of retakes so as to assess the students fully, getting them prepared for the licensing exam. Results from this may be noted in the context of Mzuzu University, which has had over 98% pass rates during licensure examinations for the past 5 years. On the other hand, there is equally a possibility that support structures are in place but are less effective, resulting in higher academic challenges for the students. Several studies have highlighted that variations in failure rates across institutions can be linked to differences in the intensity of clinical training, the stringency of assessment criteria, and the availability of academic support (Homer, 2022; McManus et al., 2006). Research further suggests that institutions with robust support systems and feedback mechanisms tend to have lower failure rates among medical students, Institutions with strong support systems, including tutoring and feedback, were found to have lower failure rates (Ajjawi et al., 2023), emphasizing the importance of a supportive academic environment in reducing failure rates among medical students(Ajjawi et al., 2023; Ellaway et al., 2018b).

### Gender and Academic Performance

Indeed, the study found that, across most institutions and in the aggregate, males were more likely to experience retakes than females. The literature supports this finding, with previous studies demonstrating gender differences in medical and health-related academic performances, including the following:

### Differences in Study Habits and Academic Support

Some studies suggest that female students may exhibit more diligent study habits and seek academic support more frequently than male students (Hesse et al., 2023). However, the impact of these differences on academic performance is unclear. A study at Augusta University found that while a gender gap in performance existed early in the curriculum and on the first national licensing exam, this gap disappeared by the end of the program and on subsequent exams (Hesse et al., 2023). This suggests that factors like active learning pedagogy may help close performance gaps between genders.

Research concluded that female medical students outperforming their male counterparts may be attributed to several factors including higher levels of conscientiousness, academic engagement, and a greater propensity to seek help during academic challenges (Li et al., 2021), Additionally, societal expectations and pressures may drive female students to excel academically in traditionally male-dominated fields, such as medicine (Carr PL, 2018). Studies show that female students exhibit better attendance and performance in formative assessments compared to males as was noted in subjects like biochemistry, where they scored significantly higher in both theory and practical assessments (Raghavani et al., 2020). Female students further report moderate preparedness to care for patients (Henrich et al., 2008; Saxena et al., 2024). This proactive approach to learning and support-seeking behavior contributes to their lower retake rates in medical education. Overall, these findings highlight the importance of addressing educational gaps while recognizing the strengths that female students bring to medical training and the weaknesses of male students.

### Bias

There is evidence suggesting that gender bias exists in medical training, particularly regarding the treatment of male and female students by lecturers. Research indicates that male lecturers may exhibit a tendency to favor female students, providing them with more attention and support during their education. This dynamic can lead to disparities in assessment outcomes, potentially putting male students at a disadvantage, in a study to investigate the experience of medical students during a clinical attachment in obstetrics and gynecology (O&G) Male students reported a significantly higher proportion of discrimination against their gender by medical officers (*p* = 0.018) and specialists/consultants (*p* < 0.001) compared to females but there was no discrimination between genders by staff nurses or house officers (Zahid et al., 2015). While there are positive strides being made towards inclusivity in medical education, significant challenges remain regarding gender bias in student support and assessment outcomes. This could be one of the causes of increased proportions of male students with retakes Addressing these issues is crucial for ensuring that all medical students receive equitable training and opportunities for success.

### Existing support systems for females

The increase in concerns is that support systems, most of which focus on females and forget the needs of males, can create a grotesque imbalance. This has far-reaching effects on performance and well-being, each important in its own right. Society traditionally expects men to embody strength and independence, leading to a reluctance among young males to express vulnerability or seek assistance. This expectation can create a stigma around mental health issues, making it difficult for the males to acknowledge their struggles or pursue help without feeling weak (McKenzie et al., 2022).

### Age and Retake Likelihood

Age appeared as a factor, which influenced the possibility of retakes. Students aged 25 and above are more likely to fail than their younger rivals. This study gave further evidence that as the age increases, the likelihood of having a retake also increases. This might relate to the fact that older students usually have additional responsibilities, like families or jobs, which could limit the time that would be put into the studies (Nalova & Etomes, 2019; Wambugu & Emeke, 2019).

Additionally, older students often face longer gaps between their earlier education and current studies, leading to challenges in adapting to new curricula and academic expectations (Nalova & Etomes, 2019). Previous research indicates that older students, especially in medical education, report higher stress levels and difficulties managing academic workloads (Nalova & Etomes, 2019; Tadese et al., 2022).

However, there is an evident gap in the literature that directly addresses the age and academic failure relationship in Sub-Saharan Africa; thus, this could call for further interrogation on the influence of culture, support systems, and comparative studies to develop an understanding that best addresses support for older learners.

### Evaluation method contributing to Academic Failure

Understanding the evaluation methods that contribute to academic failure is crucial for developing effective educational strategies. Various studies and frameworks have identified key factors and methodologies that can lead to academic challenges among students. Below are some prominent evaluation methods and their implications for academic performance

The Objective Structured Clinical Examination, OSCE, which has been reportedly failed by students, contributed to 47.7% to the overall tally of failures. This agrees with the literature, which refers to OSCEs as one of the most difficult and extensive clinical skills assessments method because of their high-stakes nature and the emphasis on clinical performance under time constraints. OSCEs involves the students in demonstrating clinical skills in a simulated environment and are often prone to stress and anxiety that impairs performance. However, it has to be taken into consideration that in this study the mode of evaluation used mostly by majority of the institutions was OSCE thus this could have led to the seen high contribution to the retakes, as such focus should be put at improving and administering transparent assessments to students so as to ensure efficient and transparent assessment of students.

OSCE enjoys wide usage as an assessment tool in medical education but is fraught with formidable challenges for students and may lead to failures and repeated takes. For improvement in assessments, medical schools have to work on the lines of better preparation of students, building support systems, and sorting out organizational challenges related to these assessments. (Alkhateeb et al., 2022; Ataro et al., 2020; Shorbagi et al., 2022).

## Conclusion

The present study contributes to an understanding of the factors influencing retake rates among health professions students in Sub-Saharan Africa and calls for institutional interventions targeting at-risk populations: older and male students, and those with assessment problems. Amelioration of these factors through appropriate support systems could minimize the rates of students having to retake modules and create a more supportive academic environment. Further research might focus on the longitudinal effect of retakes on career development for the most helpful remediation.

## Supplementary Material

1
