# Supplementary material for: Factors associated with retakes in health Professions Courses: A case study of Five selected Universities in Sub-Saharan Africa
Source: Res Sq. 2024 Nov 19:rs.3.rs-5368416. Preprint. [Version 1] doi: 10.21203/rs.3.rs-5368416/v1 (PMC11601863; doi:10.21203/rs.3.rs-5368416/v1)
Supplement: 1 [file NIHPPRS5368416v1-supplement-1.pdf]

Tables

Tables 1 to 6 are available in the Supplementary Files section

Supplementary Files

This is a list of supplementary files associated with this preprint. Click to download.

- Tables.docx
